# Transanal endoscopic microsurgery versus endoscopic mucosal resection for large rectal adenomas (TREND-study)

**DOI:** 10.1186/1471-2482-9-4

**Published:** 2009-03-13

**Authors:** Frank JC van den Broek, Eelco JR de Graaf, Marcel GW Dijkgraaf, Johannes B Reitsma, Jelle Haringsma, Robin Timmer, Bas LAM Weusten, Michael F Gerhards, Esther CJ Consten, Matthijs P Schwartz, Maarten J Boom, Erik J Derksen, A Bart Bijnen, Paul HP Davids, Christiaan Hoff, Hendrik M van Dullemen, G Dimitri N Heine, Klaas van der Linde, Jeroen M Jansen, Rosalie CH Mallant-Hent, Ronald Breumelhof, Han Geldof, James CH Hardwick, Pascal G Doornebosch, Annekatrien CTM Depla, Miranda F Ernst, Ivo P van Munster, Ignace HJT de Hingh, Erik J Schoon, Willem A Bemelman, Paul Fockens, Evelien Dekker

**Affiliations:** 1 Dept of Gastroenterology & Hepatology, Academic Medical Centre, Amsterdam, The Netherlands; 2 Dept of Surgery, IJsselland Hospital, Capelle aan de IJssel, The Netherlands; 3 Dept of Clinical Epidemiology, Biostatistics and bioinformatics, Academic Medical Centre, Amsterdam, The Netherlands; 4 Dept of Gastroenterology, Erasmus Medical Centre, Rotterdam, The Netherlands; 5 Dept of Gastroenterology, St Antonius Hospital, Nieuwegein, The Netherlands; 6 Dept of Surgery, Onze Lieve Vrouwe Gasthuis, Amsterdam, The Netherlands; 7 Dept of Surgery, Meander Medical Centre, Amersfoort, the Neterhlands; 8 Dept of Gastroenterology, Meander Medical Centre, Amersfoort, the Neterhlands; 9 Dept of Surgery, Flevoziekenhuis, Almere, The Netherlands; 10 Dept of Surgery, Slotervaart Hospital, Amsterdam, The Netherlands; 11 Dept of Surgery, Medical Centre Alkmaar, Alkmaar, The Netherlands; 12 Dept of Surgery, Diakonessenhuis, Utrecht, The Netherlands; 13 Dept of Surgery, Medical Centre Leeuwarden, Leeuwarden, The Netherlands; 14 Dept of Gastroenterology, University Medical Centre, Groningen, The Netherlands; 15 Dept of Gastroenterology, Medical Centre Alkmaar, Alkmaar, The Netherlands; 16 Dept of Gastroenterology, Medical Centre Leeuwarden, Leeuwarden, The Netherlands; 17 Dept of Gastroenterology, Onze Lieve Vrouwe Gasthuis, Amsterdam, The Netherlands; 18 Dept of Gastroenterology, Flevoziekenhuis, Almere, The Netherlands; 19 Dept of Gastroenterology, Diakonessenhuis, Utrecht, The Netherlands; 20 Dept of Gastroenterology, IJsselland Hospital, Capelle aan de IJssel, The Netherlands; 21 Dept of Gastroenterology, Leiden University Medical Centre, Leiden, The Netherlands; 22 Dept of Surgery, IJsselland Hospital, Capelle aan de IJssel, The Netherlands; 23 Dept of Gastroenterology, Slotervaart Hospital, Amsterdam, The Netherlands; 24 Dept of Surgery, Jeroen Bosch Hospital, 's-Hertogenbosch, The Netherlands; 25 Dept of Gastroenterology, Jeroen Bosch Hospital, 's-Hertogenbosch, The Netherlands; 26 Dept of Surgery, Catharina Hospital, Eindhoven, The Netherlands; 27 Dept of Gastroenterology, Catharina Hospital, Eindhoven, The Netherlands; 28 Dept of Surgery, Academic Medical Centre, Amsterdam, The Netherlands

## Abstract

**Background:**

Recent non-randomized studies suggest that extended endoscopic mucosal resection (EMR) is equally effective in removing large rectal adenomas as transanal endoscopic microsurgery (TEM). If equally effective, EMR might be a more cost-effective approach as this strategy does not require expensive equipment, general anesthesia and hospital admission. Furthermore, EMR appears to be associated with fewer complications.

The aim of this study is to compare the cost-effectiveness and cost-utility of TEM and EMR for the resection of large rectal adenomas.

**Methods/design:**

Multicenter randomized trial among 15 hospitals in the Netherlands. Patients with a rectal adenoma ≥ 3 cm, located between 1–15 cm ab ano, will be randomized to a TEM- or EMR-treatment strategy. For TEM, patients will be treated under general anesthesia, adenomas will be dissected en-bloc by a full-thickness excision, and patients will be admitted to the hospital. For EMR, no or conscious sedation is used, lesions will be resected through the submucosal plane in a piecemeal fashion, and patients will be discharged from the hospital. Residual adenoma that is visible during the first surveillance endoscopy at 3 months will be removed endoscopically in both treatment strategies and is considered as part of the primary treatment.

Primary outcome measure is the proportion of patients with recurrence after 3 months. Secondary outcome measures are: 2) number of days not spent in hospital from initial treatment until 2 years afterwards; 3) major and minor morbidity; 4) disease specific and general quality of life; 5) anorectal function; 6) health care utilization and costs. A cost-effectiveness and cost-utility analysis of EMR against TEM for large rectal adenomas will be performed from a societal perspective with respectively the costs per recurrence free patient and the cost per quality adjusted life year as outcome measures.

Based on comparable recurrence rates for TEM and EMR of 3.3% and considering an upper-limit of 10% for EMR to be non-inferior (beta-error 0.2 and one-sided alpha-error 0.05), 89 patients are needed per group.

**Discussion:**

The TREND study is the first randomized trial evaluating whether TEM or EMR is more cost-effective for the treatment of large rectal adenomas.

**Trial registration number:**

(trialregister.nl) NTR1422

## Background

Rectal cancer is a common disease in the Netherlands with approximately 4,000 new cases and 2,000 deaths annually[1]. The incidence of rectal cancer increases with age, male sex and obesity, without ethnic preference [2-4]. In the pathogenesis, premalignant intraepithelial neoplasia that is located in a rectal adenoma, precedes the occurrence of invasive rectal cancer[5,6]. Early endoscopic detection and removal of rectal adenomas prevents the development of rectal cancer and is therefore the most reliable contributor to the 'cure' of this disease[7,8]. When rectal adenomas become large, however, standard endoscopic therapies like simple loop polypectomy or one-step endoscopic resection will be inadequate. Therefore, large rectal adenomas must be removed either surgically or by extended endoscopic mucosal resection (EMR)[9].

In 1984 a novel surgical approach for the resection of large rectal adenomas has been introduced in Germany: transanal endoscopic microsurgery (TEM)[10]. This procedure encompasses general anesthesia, the use of expensive specialized equipment, a full-thickness rectal wall excision and hospital admission[11,12]. Since its introduction, many surgical practices (including the Netherlands) have adopted TEM as the new standard therapy for large rectal adenomas[13,14]. Alongside the introduction and refinement of TEM for rectal adenomas, advanced endoscopic therapies like extended EMR have rapidly evolved[15,16]. For extended EMR no sedation, no sophisticated equipment and no hospital admission are required as opposed to TEM[9]. Furthermore, only the neoplastic mucosa is resected instead of the full-thickness rectal wall, having a potential benefit of fewer complications.

Supporters of the TEM technique praise the excellent exposure of the rectum and the minimal invasiveness, as opposed to conventional surgical techniques [17-19]. Besides, recurrence rates after TEM appear to be lower when compared to conventional surgical transanal excision[20]. The TEM technique has shown to be highly efficacious in several retrospective and prospective case series with reported recurrence rates of 0–19% and complication rates of 2–21% [21-41].

On the other hand, extended EMR has gained more and more support in the last few years, mainly due to good clinical results after EMR in the esophagus and stomach[42,43]. Endoscopic mucosal resection has also been described for the treatment of large colorectal adenomas, revealing recurrence rates of 0–9% and complication rates of only 0–9% [44-53]. In case adenomas can not be removed completely during one EMR attempt, repeat EMR for residual disease generally leads to an overall success rate of 96–100%. Recently, the first prospective study analyzing extended EMR for large rectal adenomas has been described, revealing a recurrence rate of 8% and complication rate of 8%[9]. In this study, all recurrences were detected during the first control endoscopy after 3 months; repeat EMR of residual disease led to an overall success rate of 98.4%.

Since the efficacy of extended EMR for large rectal adenomas appears to be comparable to TEM, we started a prospective registration of patients with large non-pedunculated rectal adenomas who were treated by EMR in the Academic Medical Center Amsterdam. Preliminary results of this study were published in abstract form, demonstrating that EMR is safe and effective for the resection of large rectal adenomas having an overall success rate so far of 100%[54].

Until now, TEM and EMR have never been formally compared, and no such comparative studies have been registered at this moment. Although selection bias inevitably exists in prospective and retrospective case series, the results of these studies suggest that both TEM and EMR have comparable recurrence rates. Even when recurrences occur after TEM or EMR, most of these can successfully be re-treated without the need for radical surgery. The literature furthermore suggests that EMR is associated with fewer complications, reduced hospital admission, and no general anesthesia is required for EMR, all of which are favorable in both patients' and societal perspective. These contrasts of the two procedures might well lead to differences in costs and quality of life. Therefore, we designed a multicenter randomized trial to compare TEM and EMR for the resection of large rectal adenomas. The main objective of this study will be a cost-effectiveness and cost-utility analysis of these two procedures.

## Methods/Design

### Hypothesis

Transanal endoscopic microsurgery and extended EMR are both effective treatments for large rectal adenomas with comparable recurrence rates. However, EMR does not require general/spinal anesthesia or hospital admission and may be associated with lower morbidity. Therefore, EMR may improve quality of life and reduce health care costs.

### Objective

The main objective of the proposed randomized study is to compare the cost-effectiveness and cost-utility of TEM and EMR for the removal of large rectal adenomas. For a cost-effectiveness and cost-utility analysis the following study aims are taken into consideration:

▪ Comparison of recurrence rates after removal of large rectal adenomas by TEM or EMR.

▪ Comparison of morbidity and mortality associated with both procedures, by counting the number of patients with complications and the number of days that patients are alive, outside the hospital and without recurrence.

▪ Comparison of general and disease specific quality of life of patients before and after treatment by TEM or EMR.

▪ Comparison of health care service costs, production loss, and out-of-pocket expenses for TEM and EMR.

### Design

**Figure 1:**
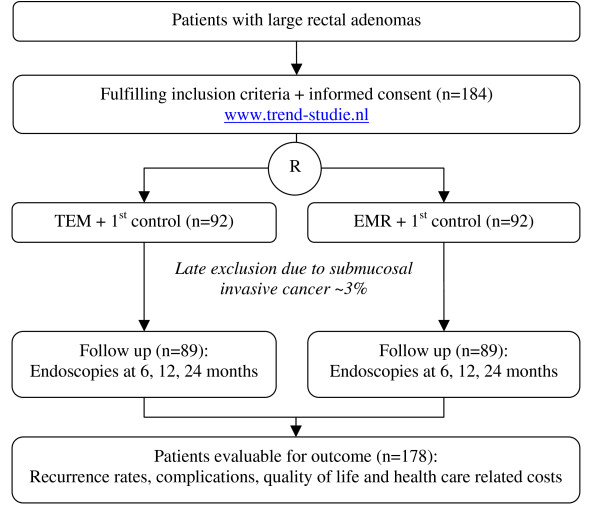
**Flow chart of TREND study; 15 Dutch centers will be participating in this multicenter randomized trial (R = randomization)**.

This will be a multicenter randomized trial comparing TEM and EMR in patients with large rectal adenomas with respect to cost-effectiveness and safety (figure 1).

### Randomization

Patient data are entered into a computerized database and by means of an unchangeable computer generated number patients will be randomized to undergo TEM or EMR. Randomization will be stratified by whether patients have a primary adenoma or residual/recurrent disease after prior resection.

### Blinding

Blinding of patients and physicians during treatment is unfeasible, since the two treatment strategies are highly different in nature and in associated care. Endoscopic follow-up for recurrence, however, will be performed by independent endoscopists who are blinded for treatment strategy (see also primary outcome measure).

### Study population

Patients are eligible for this trial when they meet the following inclusion criteria:

(1) Diagnosed with a large non-pedunculated rectal adenoma (sessile or flat) with a largest diameter of ≥ 3 cm (estimated by an opened biopsy forceps of 8 mm or an opened resection snare of 13, 20 or 30 mm).

(2) The lower border of the adenoma is located at ≥ 1 cm and ≤ 15 cm from the anal verge, respectively.

(3) Biopsies of the lesion, if taken, did not show invasion of neoplastic tissue in the submucosal layer on histopathological evaluation; only lesions with intraepithelial (low or high grade) or intramucosal neoplasia are suitable for inclusion.

(4) During flexible video endoscopy there are no signs of endoscopic suspicion for submucosal invasive cancer (Kudo pit pattern type V; excavated/depressed type morphology; fold convergence; or large smooth nodule > 1 cm in a flat lesion)[55,56].

(5) In case of doubt, endoscopic ultrasonography (EUS) of the rectal adenoma should exclude invasion into the submucosal layer and exclude pathological lymphadenopathy (lymph nodes > 1 cm). When pathological lymph nodes are present, fine needle aspiration will be performed to exclude lymph node metastasis.

(6) If not performed already, total colonoscopy will be done to detect and remove all synchronous colonic adenomas or cancers first. Cecal intubation must be confirmed by identification of the appendiceal orifice and ileocecal valve.

(7) The general health condition of the patient permits general/spinal anesthesia (ASA-classification I-III).

(8) Absence of non-correctable coagulopathy (international normalized ratio > 2, or platelet count < 90 × 10^9^/l).

(9) Patient age of 18 years or older.

### Participating centers

Fifteen Dutch hospitals of the TREND-study group, including four academic and eleven non-academic centers, will enroll patients.

### Intervention strategies

#### Transanal endoscopic microsurgery

TEM will be performed as described by Buess[12]. Under general/spinal anesthesia a specialized TEM rectoscope of 12 or 20 cm in length (Wolf GmbH Knittlingen or Storz GmbH Tuttlingen, Germany) is inserted within the rectum to assure proper visualization of the lesion. The rectoscope is fixed to the operating table by a supporting device, providing the opportunity to reposition the rectoscope during ongoing surgery. The rectal cavity is insufflated with CO_2 _by a combined endosurgical unit to achieve constant distension for appropriate visualization of the rectal adenoma. The combined endosurgical unit further regulates irrigation and suction, thereby maintaining a constant intra rectal pressure. With the use of a binocular stereoscopic eyepiece for three-dimensional view (Wolf GmbH only) or a forward oblique telescope (Storz GmbH) a magnified view is being created for visualization of the lesion. With various instruments (multifunctional TEM400 (Erbe Elektromedizin GmbH, Tubingen, Germany), Ultracision harmonic scalpel (Ethicon Endo-Surgery, Cincinnati, USA), needle diathermy, tissue handling forceps, needle holder, suction probe, injection needle, clip applicator) the adenoma will be dissected by means of an *en-bloc *full-thickness rectal wall excision until the perirectal fat. Postoperatively, patients will preserve a urinary catheter that will be removed at the first postoperative day. Patients are admitted to the hospital in accordance with current practice.

After 3 months a control flexible endoscopy will be performed. If presumed residual disease is seen, biopsies will be taken to confirm the presence of neoplasia by histology. Hereafter, the residual adenomatous tissue will be resected endoscopically by either EMR (if > 5 mm) or argon plasma coagulation (APC) (if < 5 mm). Any intervention by EMR/APC at 3 months is part of the TEM treatment strategy.

#### Endoscopic mucosal resection

Endoscopic mucosal resection is performed as described by Karita and Hurlstone[9,15]. At the discretion of the endoscopist, conscious sedation is used with 2.5–10 mg midazolam and/or 25–100 μg of fentanyl. An endoscope (gastroscope or sigmoidoscope) is inserted into the rectum and air insufflation via the endoscope will provide proper distension of the rectum. The submucosa underneath the lesion will be injected through an endoscopic injection catheter with a solution of saline 0.9%, 1 ml methylene blue, and 1:10,000 units adrenaline in order to lift the adenoma (no upper volume limit). A (barbed or standard) resection snare will be placed around a part of the lesion and subsequently tightened for resection through the submucosal layer by electro-coagulation. Each part of the adenoma will be resected by this piecemeal fashion until the entire lesion is macroscopically removed and the blue colored submucosa is visualized. Visible submucosal vessels will be treated by endoscopic clips or electro-coagulation to prevent delayed bleeding. Hereafter, the edges of the mucosal defect and potential remnants within the resection crater will always be treated with APC to increase adenoma clearance. If bleeding during the procedure precludes 100% clearance of the adenoma, the EMR procedure will be continued after 1 day until all adenomatous tissue is resected. In case of procedural blood loss of > 100 mL or if delayed perforation is anticipated on procedural grounds, patients are admitted to the hospital for observation; otherwise, they are discharged after the procedure in accordance with current practice.

After 3 months the treating endoscopist will perform a control endoscopy. If presumed residual disease is seen, biopsies will be taken to confirm the presence of neoplasia by histology. Hereafter, the residual adenomatous tissue will be resected endoscopically by either EMR (if > 5 mm) or APC (if < 5 mm). Any intervention by EMR/APC at 3 months is part of the EMR treatment strategy.

### Informed consent procedure

Consecutive eligible patients will be recruited at the outpatient clinic in the participating centers by the involved physician (surgeon or gastroenterologist). Patients fulfilling the abovementioned inclusion criteria will be informed about the study by the physician. After written informed consent, patients will be allocated to either TEM or EMR by computerized block randomization with variable block size via the study website . The patient will subsequently be scheduled for therapy in the participating centre. Patients unable or refusing to provide informed consent will be treated according to current clinical practice.

### Intervention failure

When for technical reasons EMR procedures turn out not to be performable after randomization, the patient will automatically undergo the TEM treatment strategy. When TEM turns out not to be performable, an attempt with EMR will be done as well. When adenomas turn out to be too large or too high in the rectum for TEM by the Storz equipment, the patient will undergo TEM in another hospital where Wolf equipment is available.

### Safety monitoring

In order to control for quality of the treatment (TEM or EMR) all procedures will be taped for reference. The first 3 procedures and an additional random sample per specialist will be assessed for quality by an expert panel at 2 time points during each year.

### Histopathological evaluation

Resection specimens after TEM will be stretched and pinned on a cork plate before immersion into formalin. After standard processing the resection specimen will be transected each cm for evaluation by a gastrointestinal pathologist. The lateral and basal resection margins will be evaluated for absence of neoplasia, when possible. All resected pieces by EMR will be processed in the same manner, and only the basal resection margins will be evaluated.

The risk of lymph node metastases is increased in case of neoplasia extending into the submucosal layer, poor tumor differentiation, mucinous cancer, vascular invasion and tumor budding, all of which warrant further radical surgery [57-60]. By strict adherence to the inclusion criteria, the risk of invasive cancer is reduced to 1.6–3%[56,61]. In case of an unexpected invasive cancer despite adherence to the inclusion criteria, the patient will be excluded after the histopathological evaluation (late exclusion). In case of intramucosal cancer (i.e. not invading through the muscularis mucosae), both TEM and EMR will be regarded as sufficient treatment when the lesion is radically removed.

### Outcome parameters

#### Primary outcome measure (for non-inferiority)

(1) Recurrence of neoplasia, defined as the presence of histologically proven neoplastic tissue in either visible recurrent lesions or in random biopsies, taken at surveillance endoscopies after the intervention strategy has been completed.

Any remnant adenoma identified and treated by EMR/APC at 3 months is considered part of the initial intervention strategy in both arms. Hereafter, patients will undergo surveillance endoscopies (with a GIF-Q160 endoscope) at 6, 12 and 24 months by an independent endoscopist who is blinded for the primary treatment. During each surveillance endoscopy recurrence will objectively be defined by the Higaki criteria for recurrence: tumor appearing within a clear resection scar; tumors with convergent folds; and tumors nearby a clear resection scar (within 5 mm)[49]. Targeted biopsies will be taken for histological confirmation; in case of an apparently healed normal scar without evidence of recurrence, 3 biopsies will be taken from the basis and 3 from the edges of the scar to detect occult recurrent neoplasia.

(2) Since equal recurrence rates for the TEM and EMR strategies are anticipated (equivalence trial), an additional outcome measure has been chosen that is responsive to both differences in initial care and to additional procedures that may be required in both strategies: the number of days that a patient is alive, outside the hospital and free of recurrence during two-year follow-up starting at the day of the initial treatment. Every patient therefore has potentially 730 days, and hospital days will be subtracted for initial treatment, readmissions, re-interventions and surveillance endoscopies. Adenoma recurrence or death will be considered as failure of the treatment strategy and no more additional days outside the hospital will be counted for such a patient.

'Unrelated' readmissions may have a relatively large impact on the number of days outside the hospital as well as on costs in this equivalence trial, if by chance they are unevenly distributed among treatment arms. Therefore, we will exclude the unrelated readmissions in a subsequent sensitivity analysis (both *clinically *and during the *cost-effectiveness and cost-utility analyses*), meaning that days in hospital due to clearly unrelated causes will not be subtracted from the total number of potential days outside the hospital for a patient. Likewise, other unrelated health care or unrelated days of sick leave will be excluded in this sensitivity analysis. Whether readmissions are related or unrelated to the target condition will be assessed by an independent expert panel (blinded for treatment).

The following standardized discharge criteria will be applied in all participating hospitals: normal intake of nutrition; normal mobility; absence of fever (< 38°C); and stable hemoglobin level during 1 day (< 1 mmol/L) in case of rectal blood loss.

#### Additional outcome measures

(a) Complications: subdivided into procedural (during treatment) and delayed complications (after ending the procedure); and further subdivided into major (requiring additional surgery) and minor (requiring endoscopic or medical intervention) complications.

During admission patients will be monitored for complications. In case of same day discharge from the hospital patients will be called by telephone 1 day after the procedure whether adverse events have happened. Two weeks after the intervention, a research nurse will contact the patient by telephone again and ask for occurred complications.

(b) Generic and disease-specific health related quality of life will be measured at baseline, 2 weeks, 3 months, 6 months, 1 year and 2 year follow-up by the EQ-5D, SF-36, Wexner score (for incontinence) and COREFO questionnaires[62].

(c) Costs of TEM and EMR from a societal perspective, based on primary data (see economic evaluation section).

(d) Patient preferences regarding TEM or EMR will be measured at the end of follow-up by a structured questionnaire to enable a discrete choice experiment addressing the burden of care, burden of complications, prognostic uncertainties, and recurrence rates of both treatments.

### Sample size calculation

Assuming a baseline recurrence rate of 3.3% for both TEM and EMR (average recurrence based on a systematic review) and considering an upper limit of 10% for EMR to be non-inferior, with a β-error of 0.2 and α-error of 0.05, 89 patients are needed per randomization group. As unexpected invasive cancers are expected in maximally 3% of patients, the total sample size will be 184 patients.

Since EMR is known to be effective in even more than 2 attempts, an upper limit of 10% seems reasonable, whereas higher recurrence rates would lead to many additional procedures which renders this strategy impracticable and probably not cost-effective.

### Ethics

This study is conducted in accordance with the principles of the Declaration of Helsinki and 'good clinical practice' guidelines. The medical ethical committee of the Academic Medical Centre Amsterdam has approved the study protocol (MEC number 08/183 # 08.17.1104). Prior to randomization, written informed consent will be obtained from all patients.

### Data-analysis

Since the main outcome of this study is the neoplasia recurrence rate, i.e. proportion of patients with recurrent disease, the Chi-square test will be used to compare the intervention groups (TEM versus EMR). Since the event of recurrence, and not time to recurrence, is the most important indicator for treatment failure, Kaplan Meier methods will not be used. The complication and mortality rates will be compared in the same manner. The number of days not spent in hospital as additional primary outcome measure will be compared by the Wilcoxon rank sum test.

Differences between the intervention groups in continuous outcome measures (e.g. Wexner incontinence scale, quality of life questionnaires) will be tested by the student's t-test or Wilcoxon rank sum test, where appropriate.

All analyses will be carried out primarily on an intention-to-treat basis.

### Economic evaluation

#### General considerations

The economic evaluation of EMR against TEM for large rectal adenomas will be performed as a cost-effectiveness analysis as well as a cost-utility analysis from a societal perspective. The primary outcomes are the costs per recurrence free patient and the costs per quality adjusted life year respectively. The costs per patient free of complications as well as the costs per day alive and outside the hospital will be considered as secondary outcome. The time horizon is restricted to a follow-up of 24 months. Given this time span, discounting (of costs and effects) will be performed. Incremental cost-effectiveness ratios are calculated, reflecting the extra costs per additional recurrence free patient and the extra costs per additional QALY. Sensitivity analyses will be performed to account for sampling variability (following bias corrected and accelerated non-parametric bootstrapping), for plausible ranges in unit costs of surgery and endoscopic treatment, for (differential) discount rates of costs and effects, and for different health utility algorithms (see below). Subgroup analyses will be performed for patients with different rectal adenoma diameters (< 5 cm, 5–10 cm, > 10 cm) and distances of the adenoma from the anal verge (< 7.5 cm versus 7.5–15 cm) in order to tentatively assess differences in health care efficiency.

In case TEM and EMR turn out clinically equivalent, the study will be performed as a cost-minimization analysis[63].

#### Cost analysis

The evaluation will include the direct medical costs, out-of-pocket expenses, and the indirect non-medical costs of production loss. The direct medical costs will include the costs of all diagnostic procedures (except study-related ones like anal manometry), therapeutic (repeat) interventions, medication, admissions, day care treatments, specialist consultations, and out-of-hospital care (like general physician, fecal incontinence pads, etc) during follow-up. With approximately over 40% of patients below 65 years of age, production losses will be estimated and based on questionnaire data concerning absence from work and lower efficiency while at work. Out-of-pocket expenses will include the costs of health-related travel, over-the-counter medication, extra washing, etc. Volume data will be gathered with clinical report forms, available hospital information systems, and the Dutch Health and Labour Questionnaire (to be completed by patients at baseline, week 2, and months 3, 12, and 24). The Dutch costing guideline for health care research will be used to determine the relevant unit costs. In case of the TEM and EMR however, micro-costing (general/spinal/conscious anesthesia or sedation, surgical and endoscopic equipment, procedure duration, involved personnel, overhead) in participating centers will be done to estimate real unit costs. The friction costs method will be applied to derive the costs of lost productivity. After price-indexing all costs will be expressed in 2009 euros.

#### Patient outcome analysis

Patients' health status and quality of life will be assessed with the Wexner score, COREFO and SF-36. In addition, the EQ-5D questionnaire is used to generate health status scoring profiles over time, which will subsequently be translated in QALYs by applying time trade-off based health utility algorithms [64,65] (see also the sensitivity analyses above) and assuming that a health utility score at any point in time best reflects a patients health status in-between the actual and the previous measurement. In addition, patient preferences regarding TEM and EMR will be measured at the end of follow-up by a structured questionnaire to enable a discrete choice experiment addressing the burden of care, burden of complications, prognostic uncertainties, and recurrence rates of both treatments[66].

## Discussion

Colorectal cancer (CRC) is the second most common cancer in the Netherlands with 9,989 new cases and 4,429 deaths in the year 2003[1]. Rectal cancer accounts for approximately 40% of those CRC cases. The treatment of rectal cancer encompasses a multidisciplinary collaboration including gastroenterologists, surgeons, oncologists, radiotherapists and specialized nurse practitioners. Standard therapy consists of radical surgery in combination with radiotherapy (and possibly chemotherapy), which have major morbidity and mortality[67]. Therefore, this disease has a major impact on health care services[68]. Since early detection and removal of rectal adenomas prevents the occurrence of rectal cancer, CRC screening has been adopted in many western countries[69]. In the Netherlands a pilot study for CRC screening, based on fecal occult blood testing, has been performed (ZonMw funded)[70]. When CRC screening is introduced in the Netherlands, this will inevitably lead to an increased detection of early rectal neoplasia[71]. It is therefore expected that more rectal adenomas will need endoscopic or surgical treatment in the forthcoming years. Consequently, the most appropriate therapy concerning efficacy, safety, quality of life and costs must be selected to deal with the expected increase in rectal adenomas.

Traditionally, adenomatous colorectal lesions that could not be resected endoscopically were referred for surgery. Conventional surgical approaches like radical surgery and trans-sphincteric or trans-sacral operations have nowadays been replaced by TEM, since this procedure has higher efficacy and lower morbidity [18-20]. However, no improvement in quality of life could be encountered by TEM, when compared to conventional radical surgery[17]. In recent years endoscopic therapies have further evolved, as a result of which large rectal adenomas more often are treated endoscopically, at the expense of TEM[9]. In case series, endoscopic resection of large colorectal adenomas has led to recurrence rates that were comparable to TEM (2.0% vs. 3.6% respectively), and complication rates that appeared lower (4.4% vs. 10.7%)[9,22,23,26,27,29-31,34-37,41,45,48,49,52,53,72-74]. Furthermore, EMR can safely be performed without sedation, or with conscious sedation only, and generally no hospital admission is required as opposed to TEM. The reduced morbidity, reduced hospital admission and redundancy of anesthesia associated with EMR are beneficial from both patients' and societal perspective.

Nevertheless, current clinical practice concerning the treatment of large rectal adenomas mainly depends on experience with TEM or EMR in various hospitals and on the clinical judgment of the involved physician instead of on evidence, which is lacking with reference to the treatment of large rectal adenomas. Although the literature suggests that TEM and EMR are equivalent techniques concerning efficacy and that EMR appears favorable concerning morbidity, a formal comparison of cost-effectiveness and cost-utility has never been performed and no ongoing studies comparing these two techniques have been registered in trial-registers so far.

## Abbreviations

TREND: TRansanal ENDoscopic microsurgery versus endoscopic mucosal resection for large rectal adenomas; TEM: transanal endoscopic microsurgery; EMR: endoscopic mucosal resection; EUS: endoscopic ultrasonography; ASA: American Society of Anaesthesiologists; APC: argon plasma coagulation; EQ-5D: Euroqol 5 Dimensions; SF-36: short form-36; COREFO: COloREctal Functional Outcome questionnaire.

## Competing interests

The authors declare that they have no competing interests.

## Authors' contributions

FvdB, EdG, MD, JR, WB, PF, ED have made substantial contributions to the conception and design of this study; have been involved in drafting the manuscript (FvdB) or revising it critically for important intellectual content (EdG, MD, JR, WB, PF, ED); and have given final approval of the version to be published.

JH, RT, BW, MG, EC, MS, MB, ED, AB, PD, CH, HvD, GH, KvdL, JJ, RM-H, RB, HG, JH, PD, AD, ME, IvM, IdH, ES have made contributions to the design of this study and have made substantial contributions to the organization of this trial in several meetings; have given final approval of the version to be published; and are local investigators at the participating centers.

## Pre-publication history

The pre-publication history for this paper can be accessed here:


